# Precision Compensation and Annealing Process Exploration for Near-Net Cold Forming of Ta-2.5W Shaped Charge Liners

**DOI:** 10.3390/ma19173737

**Published:** 2026-09-02

**Authors:** Tingjun Cai, Haicheng Shi, Wentai Zhao, Bowen Pan, Hao Wu, Guiqian Xiao, Liming Gong, Guozheng Quan

**Affiliations:** 1 Liaoshen Industries Group Co., Ltd., Shenyang 110000, China; 2 Chongqing Key Laboratory of Advanced Mold Intelligent Manufacturing, College of Materials Science and Engineering, Chongqing University, Chongqing 400044, China; 3 Mingyue Lake Laboratory, Chongqing 401135, China

**Keywords:** Ta-2.5W alloy, near-net cold forming, annealing, die compensation, shaped charge liner

## Abstract

Ta-2.5W alloy is a promising liner material for high-performance shaped-charge warheads because of its high density and excellent dynamic mechanical properties. However, conventional machining and hot-forming routes suffer from low material utilization, limited dimensional accuracy, and oxidation-related defects. In this study, near-net-shape cold pressing and annealing treatments were investigated for Ta-2.5W liners. The initial microstructure and mechanical properties of the starting sheet were characterized, compression tests were performed to establish a room-temperature constitutive model, and 16 combinations of deformation and annealing temperature were designed to clarify the evolution of grain morphology and crack sensitivity. To compensate for elastic die deformation and blank springback, a coupled simulation-based die correction strategy was further developed. The results show that the starting alloy exhibits an excellent strength–ductility balance with weak anisotropy. Increasing cold deformation refines the grains, whereas increasing annealing temperature initially promotes grain refinement but subsequently causes grain coarsening. Excessive deformation combined with high annealing temperature increases crack susceptibility. Based on the single-specimen screening experiments in this study, a preliminary processing range of 20–40% cold deformation and 1200 °C annealing produced the most favorable microstructural condition without obvious cracking. After iterative die compensation, trial-manufactured parts satisfied the target contour requirements and showed uniform, crack-free microstructures after annealing.

## 1. Introduction

Shaped charge liners are critical functional components in applications such as oil and gas well completion, armor penetration, engineering blasting, and controlled demolition [1,2]. As service requirements for penetration depth, jet stability, and reliability continue to increase, tighter control over geometric accuracy, surface quality, and microstructural uniformity is required [3]. Under the coupled conditions of high strain rates and elevated temperatures, the dynamic response of the material strongly affects jet continuity and stability, which in turn drives the development of high-performance liner materials and precision manufacturing routes.

Conventional liners are commonly made of pure copper because of its excellent ductility and machinability. However, the strength and penetration capability of pure copper are no longer sufficient for demanding applications. Ta-W alloys possess a high density, high melting point and acoustic velocity, and excellent impact resistance and dynamic mechanical properties [4,5,6]. Therefore, they have been increasingly used for the fabrication of high-performance shaped charge liners [7,8,9]. Nevertheless, these alloys are difficult to machine and impose stringent requirements on forming process design and microstructure control. Current fabrication routes still rely mainly on precision turning, sheet stamping, and forging [10,11]. Mechanical machining results in low material utilization and high manufacturing costs, whereas thermal processing can introduce oxidation and grain coarsening, both of which are detrimental to surface quality and microstructural properties. Given the high cost of Ta-W alloys, improving material utilization while maintaining dimensional accuracy is a key engineering challenge. Near-net cold forming is therefore attractive because it can suppress oxidation, improve material utilization, and produce high-precision parts at relatively low temperatures [12,13,14]. Nevertheless, deformation compatibility, springback control, microstructure evolution, and defect suppression remain major challenges in cold forming.

To address these issues, several studies have explored the forming behavior and microstructural control of Ta-W alloys. Zhan et al. [15] proposed a five-pass cold extrusion-forging route, comprising three forming passes and two annealing passes, for hyperbolic shaped charge liners. They analyzed the effect of preforming on the evolution of the final microstructure and properties, optimized the key parameters through finite element simulation, and validated the process experimentally. Yang et al. [16] compared a multipass, low-pressure, reciprocating cold-rolling route with a low-pass, high-pressure, unidirectional cold-rolling route and showed that, under the same annealing conditions, the former yielded superior mechanical properties. Kedharnath et al. [17] applied multidirectional forging to a coarse-grained Ta-10W alloy and reported substantial grain refinement, a higher fraction of high-angle grain boundaries, and an increase in yield strength from 964 to 1202 MPa. The addition of tungsten nearly doubled the strength of the alloy compared with that of pure Ta, confirming the excellent mechanical potential of Ta-W alloys.

Although meaningful progress has been made in plastic forming and microstructural control of Ta-W alloys, systematic studies on the precision cold forming of complex curved liners remain limited. In particular, the coupling between the cold-deformation history and subsequent recrystallization behavior under near-net-shape forming conditions has not been fully clarified, thereby restricting the reliable manufacture of high-performance Ta-W liners. In this work, Ta-2.5W liner blanks were investigated using a combination of experiments and numerical simulations. The initial microstructure and mechanical properties were characterized, a room-temperature constitutive model was established, the effects of deformation and annealing temperature on microstructural evolution and cracking susceptibility were evaluated, and an iterative die-compensation strategy was developed to account for elastic die deformation and blank springback. This study aims to provide a practical basis for the precision near-net-shape forming of Ta-W shaped charge liners.

## 2. Materials and Methods

The chemical composition of the as-received Ta–2.5W alloy was determined using complementary analytical techniques. The W, Fe, and Ni contents were measured using an X-ray fluorescence spectrometer (model: XRF-1800, Shimadzu Corporation, Kyoto, Japan). The C content was determined using a high-frequency infrared carbon–sulfur analyzer (model: CS-8820, Wuxi Jinyibo Instrument Technology Co., Ltd., Wuxi, China), whereas the O, N, and H contents were measured using an oxygen–nitrogen–hydrogen analyzer (model: TCH-600, LECO Corporation, St. Joseph, MI, USA). The Ta content was reported as the balance. The mass fraction of tungsten was 2.47 wt.%, tantalum constituted the matrix, and the main impurity elements were C, N, O, H, Fe, and Ni, with a total impurity content less than 0.15 wt.%. The detailed measured chemical composition is listed in Table 1. To characterize the initial material state after cold rolling and recrystallization annealing and to provide input data for constitutive modeling, the initial microstructure and tensile properties of the sheet were first evaluated.

Metallographic specimens were sectioned using a wire electrical discharge machine (model: DK7732, Jiangsu Fangzheng CNC Machine Tool Co., Ltd., Taizhou, China). Subsequent grinding and polishing were performed manually. Specimens were sectioned from the as-received sheet, ground using #400, #600, #800, #1000, and #1200 abrasive papers, and mechanically polished. They were then etched using a mixed solution containing 30 vol.% sulfuric acid, 10 vol.% nitric acid, 10 vol.% hydrofluoric acid, and 50 vol.% absolute ethanol at room temperature for 30 s. After cleaning with absolute ethanol and drying in air, the microstructure was examined using an optical metallographic microscope (model: 200MAT, Carl Zeiss AG, Oberkochen, Germany).

Flat tensile specimens with a thickness of 2 mm, a parallel-section width of 20 mm, and an original gauge length of 40 mm were machined from the sheet. Room-temperature tensile tests were conducted in accordance with ISO 6892-1:2019 [18]. Tensile experiments were performed using a servo-hydraulic universal testing machine (model: UTM5305SLXY, Shenzhen Suns Technology Stock Co., Ltd., Shenzhen, China) at a constant strain rate of 0.1 s^−1^. An extensometer was installed on the specimen to collect axial and transverse strain signals. Poisson’s ratio was calculated by the ratio of transverse strain to axial strain within the elastic deformation range. Room-temperature tensile tests were performed to obtain the stress–strain response, elastic modulus, and Poisson’s ratio, thereby enabling the evaluation of the strength, ductility, and anisotropy of the alloy.

The anisotropy coefficient was calculated using $R=\varepsilon_{w}/\varepsilon_{t}$, where $\varepsilon_{w}$ and $\varepsilon_{t}$ represent the true width strain and true thickness strain, respectively. For the calculation of the R-values, data falling within the uniform-plastic-deformation regime were used, while the elastic-deformation range and after the onset of necking were excluded.

All recrystallization annealing treatments were performed in a vacuum high-temperature furnace (model: KT-VMW, Zhengzhou Kintek Solution Co., Ltd., Zhengzhou, China) under a vacuum atmosphere better than 5 × 10^−3^ Pa. Specimens were heated from room temperature to the target annealing temperature at a heating rate of 10 °C/min. The holding period started once the target temperature was reached, with a holding time of 45 min. Upon completion of the holding period, the specimens were cooled in the furnace at an average cooling rate of approximately 5 °C/min.

To systematically evaluate the effects of deformation and recrystallization annealing on microstructural evolution and susceptibility to damage, cylindrical compression specimens with a diameter of 4 mm and a height of 5 mm were prepared. The specimens were cold compressed to deformation levels ranging from 10% to 40% and subsequently annealed at temperatures ranging from 1150 to 1300 °C for 45 min. The strain rate in all compression tests was fixed at 0.1 s^−1^, and the experimental matrix is summarized in Table 2. After annealing, the specimens were ground, polished, and etched according to the same procedure described above and examined using optical microscopy.

**Figure 1:**
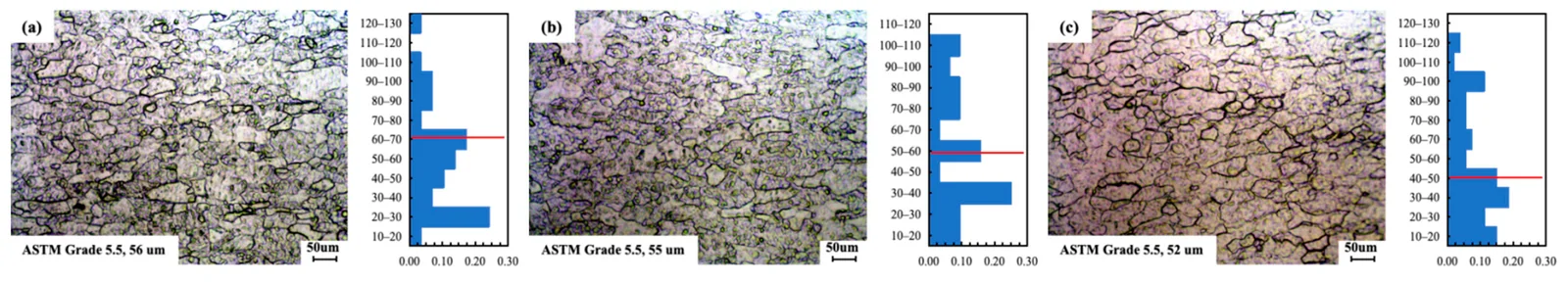
Microstructures of the initial material along different orientations at 200× magnification: (**a**) RD; (**b**) TD; (**c**) ND.

Figure 1 shows that the average grain size varies only slightly across the three observation directions, ranging from 52 to 56 μm. No significant difference in grain size is observed among the rolling direction (RD), transverse direction (TD), and normal direction (ND). The grains exhibit slight elongation, indicating a low degree of microstructural anisotropy.

## 3. Material Test Results and Constitutive Model

### 3.1. Room-Temperature Mechanical Properties

**Figure 2:**
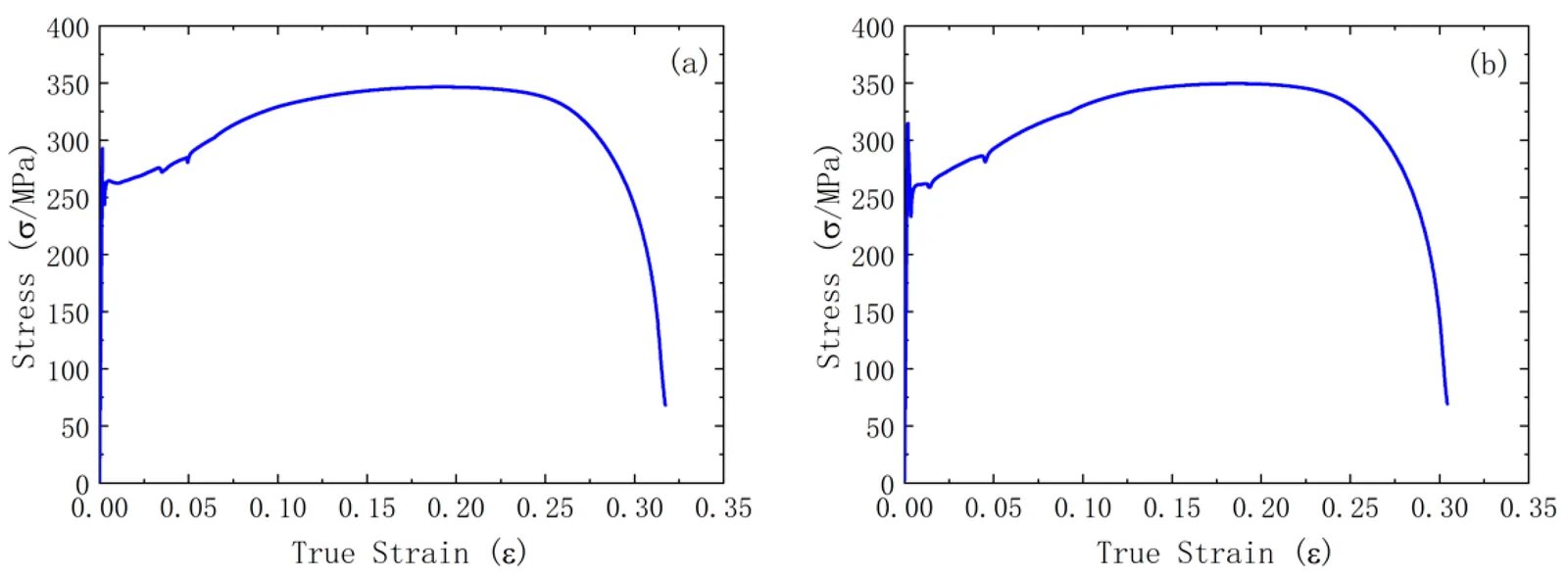
Tensile stress–strain curves along different orientations: (**a**) RD; (**b**) TD.

The room-temperature tensile stress–strain curves are presented in Figure 2. The alloy exhibits an ultimate tensile strength of approximately 350 MPa, an upper yield strength of 314.9 MPa, a lower yield strength of 233.3 MPa, and a 0.2% proof strength of 242.1 MPa. The elongation at fracture is approximately 35.5%, while the elastic modulus and Poisson’s ratio are 176 GPa and 0.28, respectively. These results indicate that the starting material achieves a favorable balance between strength and ductility. An anisotropy coefficient of approximately 0.74 indicates that the overall tensile response exhibits relatively weak anisotropy.

### 3.2. Compression Testing and Constitutive Modeling

**Figure 3:**
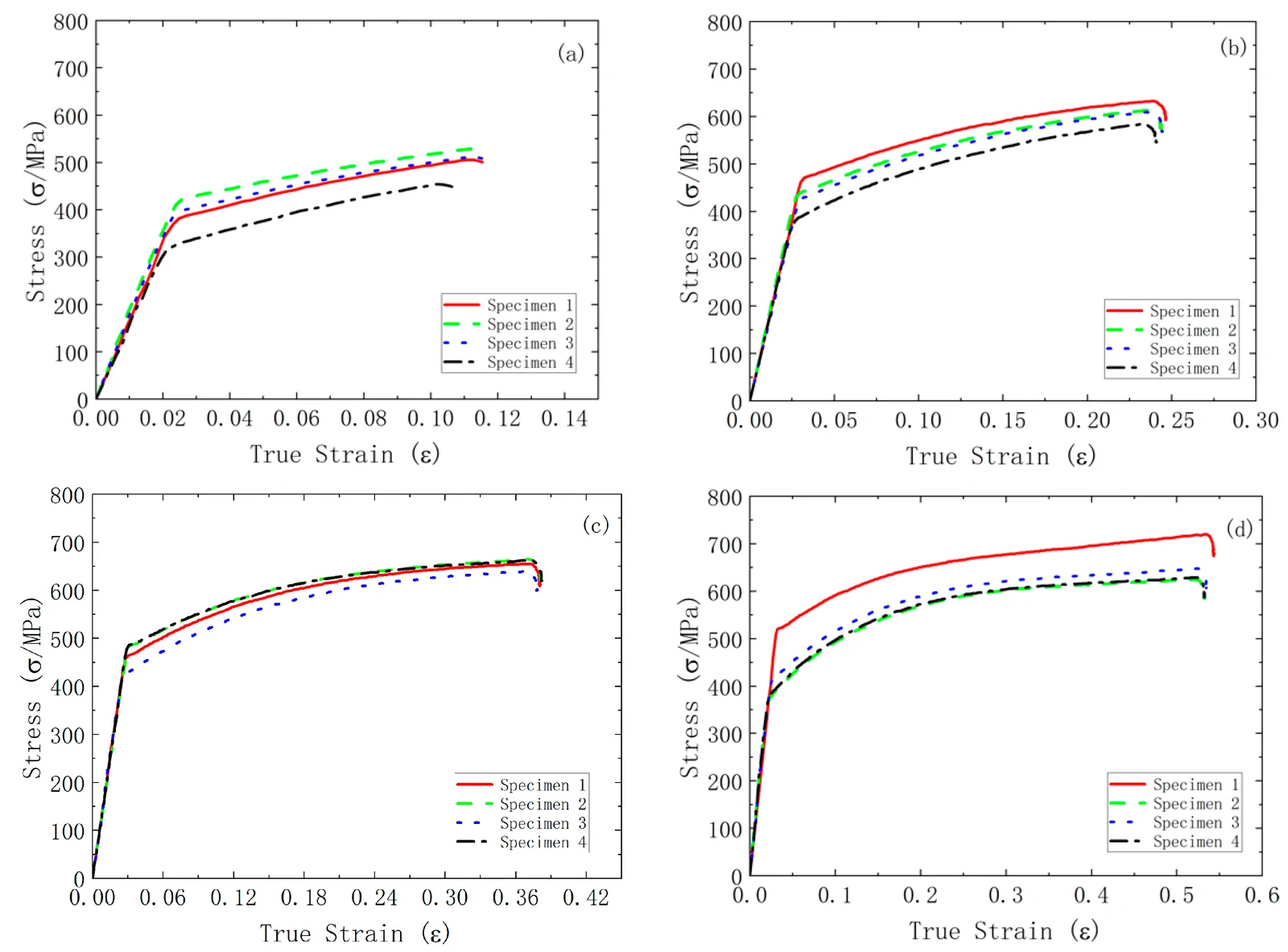
True stress–strain curves under different deformation levels: (**a**) 10%; (**b**) 20%; (**c**) 30%; (**d**) 40%.

Figure 3 shows the true compressive stress–strain curves for the 16 specimens. The curves nearly coincide in the elastic regime, indicating that the specimens exhibit consistent elastic behavior. Minor variations are observed in the plastic regime, primarily because of slight material inhomogeneity and experimental scatter. Overall, all 16 specimens demonstrate highly consistent compressive behavior and deformation characteristics.

The averaged true compressive stress–strain curves of the 16 specimens were used to establish a room-temperature constitutive model for the alloy, providing input for the cold pressing simulations. Numerical simulations of sheet cold-press forming and spring-back were performed using the dedicated cold-press forming module in Forge. The HS constitutive equation is natively embedded in this module and optimized for cold-forming applications. Experimental calibration shows that the HS model achieves high fitting precision and is capable of reproducing the plastic response of the target material. Given its good agreement with experimental measurements and its built-in optimization for cold-press conditions, the HS model was considered the most suitable candidate to represent the material mechanical behavior in the current work. The data were fitted by linear regression using the Hensel–Spittel (HS) constitutive model, and the resulting constitutive equation is given below:

(1) $$\sigma =703.76\varepsilon^{0.088}exp\left(\frac{-0.0092}{\varepsilon }\right)$$

where *σ* denotes the true stress and *ε* denotes the true strain. Combined with the measured elastic parameters, Equation (1) defines the room-temperature elastoplastic constitutive model used for cold pressing simulation.

### 3.3. Cold Compression and Recrystallization Annealing Experiments

**Figure 4:**
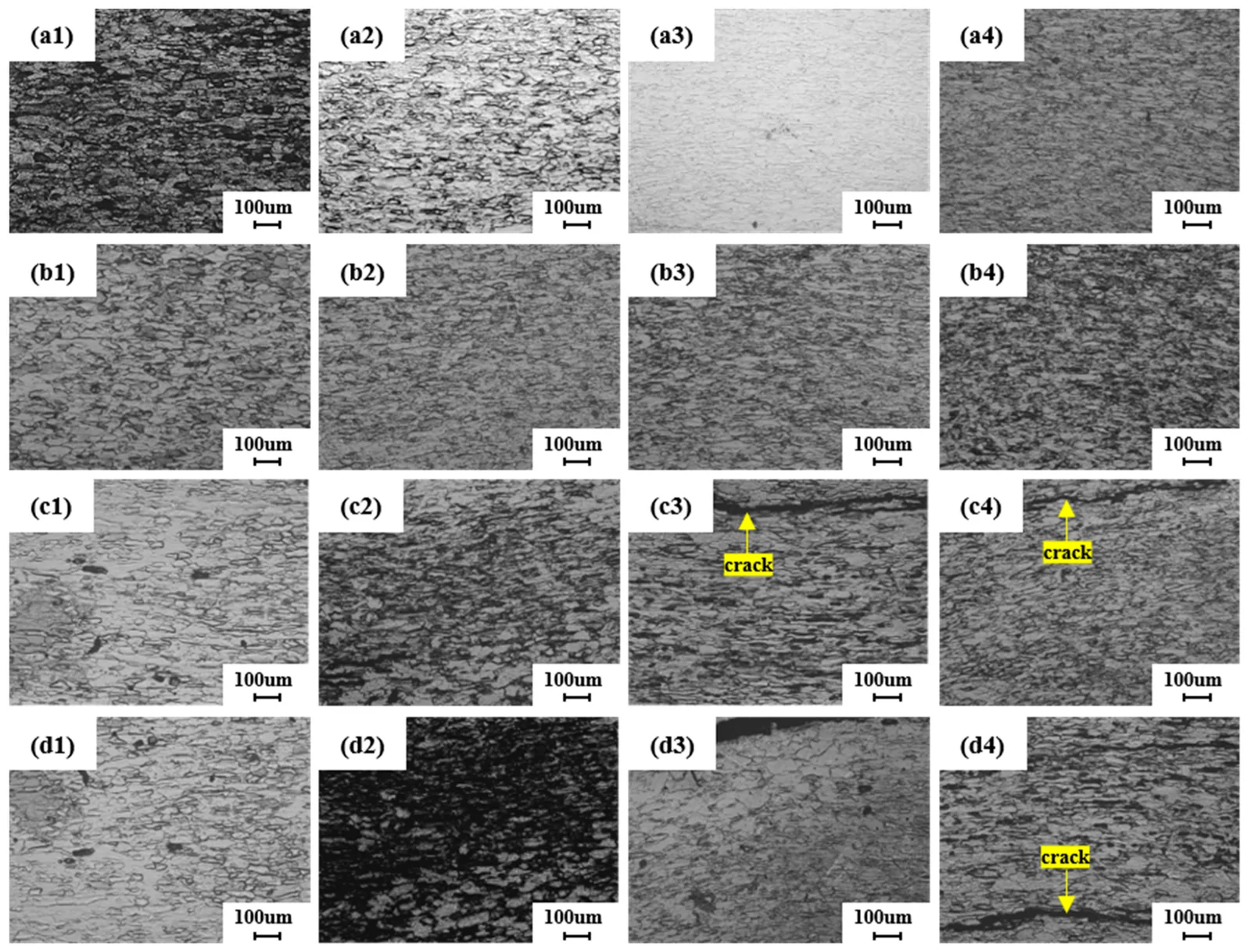
Microstructures obtained under different annealing temperatures and deformation levels at 100× magnification: (**a1**) 1150 °C, deformation 10%; (**a2**) 1150 °C, deformation 20%; (**a3**) 1150 °C, deformation 30%; (**a4**) 1150 °C, deformation 40%; (**b1**) 1200 °C, deformation 10%; (**b2**) 1200 °C, deformation 20%; (**b3**) 1200 °C, deformation 30%; (**b4**) 1200 °C, deformation 40%; (**c1**) 1250 °C, deformation 10%; (**c2**) 1250 °C, deformation 20%; (**c3**) 1250 °C, deformation 30%; (**c4**) 1250 °C, deformation 40%; (**d1**) 1300 °C, deformation 10%; (**d2**) 1300 °C, deformation 20%; (**d3**) 1300 °C, deformation 30%; (**d4**) 1300 °C, deformation 40%.

**Figure 5:**
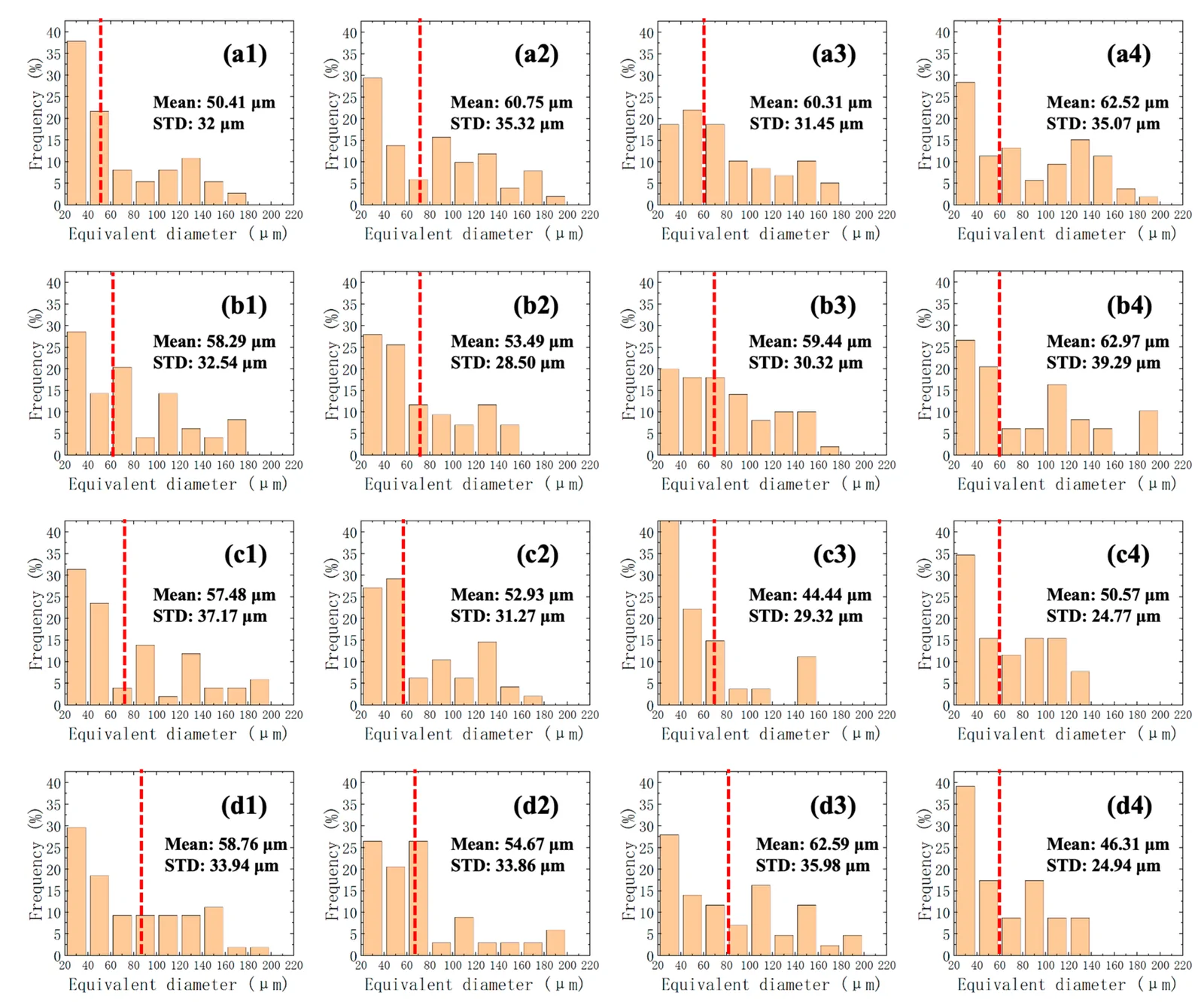
Grain-size statistical results under different annealing temperatures and deformation levels at 100× magnification: (**a1**) 1150 °C, deformation 10%; (**a2**) 1150 °C, deformation 20%; (**a3**) 1150 °C, deformation 30%; (**a4**) 1150 °C, deformation 40%; (**b1**) 1200 °C, deformation 10%; (**b2**) 1200 °C, deformation 20%; (**b3**) 1200 °C, deformation 30%; (**b4**) 1200 °C, deformation 40%; (**c1**) 1250 °C, deformation 10%; (**c2**) 1250 °C, deformation 20%; (**c3**) 1250 °C, deformation 30%; (**c4**) 1250 °C, deformation 40%; (**d1**) 1300 °C, deformation 10%; (**d2**) 1300 °C, deformation 20%; (**d3**) 1300 °C, deformation 30%; (**d4**) 1300 °C, deformation 40%.

Figure 4 and Figure 5 illustrate the influence of deformation and annealing temperature on the microstructure. At a given annealing temperature, increasing the deformation level from 10% to 40% progressively decreases the average grain size, reduces the fraction of elongated banded grains, and improves microstructural uniformity.

When the annealing temperature was 1150 °C (Figure 4(a1–a4) and Figure 5(a1–a4)), the average grain size decreased continuously with increasing deformation. At 10% deformation, the grains were relatively coarse and exhibited distinct banded structures. As deformation increased to 20%, grain refinement commenced, although a considerable fraction of elongated grains remained. At 30%, further grain refinement was achieved, but some strip-like grains persisted. At 40%, grain refinement was much more pronounced; the fraction of elongated grains was significantly reduced, and the microstructure became more homogeneous. When the annealing temperature was 1200 °C (Figure 4(b1–b4) and Figure 5(b1–b4)), a similar trend was observed. The 10% deformed specimen still contained many elongated grains after cold pressing. Raising the deformation to 20% and 30% yielded a more refined microstructure and diminished banded features. At 40% deformation, the grains were further refined, and the microstructure became comparatively homogeneous. When the annealing temperature was 1250 °C (Figure 4(c1–c4) and Figure 5(c1–c4)), grain refinement was enhanced with increasing deformation, but the tendency for crack formation became apparent. The 10% deformed specimen still exhibited relatively coarse, elongated grains. At 20% deformation, grain refinement took place, yet local crack initiation occurred. At 30%, long strip-like cracks were observed, and at 40% deformation the cracks became more pronounced even with a refined and relatively homogeneous grain structure. When the annealing temperature was 1300 °C (Figure 4(d1–d4) and Figure 5(d1–d4)), the average grain size again decreased with increasing deformation, but cracking grew more severe. The 10% deformed specimen retained many elongated grains, whereas the 20% deformed specimen exhibited more obvious grain refinement. At 30% deformation, long strip-like cracks were present, and at 40% deformation long through-thickness cracks developed.

In summary, microstructure observations from Figure 4 and Figure 5 revealed that, at a constant deformation level, increasing the annealing temperature from 1150 °C to 1300 °C first refined and then coarsened the grains. At a constant annealing temperature, increasing deformation reduced the average grain size and suppressed the original banded microstructure. Nevertheless, the combination of large deformation and high annealing temperature raised the risk of cracking. By balancing grain refinement and structural integrity, a preliminary processing range suggested by the present single-specimen screening experiments was obtained for cold deformation of 20–40%, followed by annealing at approximately 1200 °C.

## 4. Die Compensation and Experimental Validation

Owing to the high cost of Ta-W alloys, their use in liner manufacturing places stringent demands on material utilization. As a typical thin-walled component, the liner is prone to springback during cold pressing, while the die undergoes elastic deformation under the forming load. These coupled deformation effects reduce dimensional accuracy and increase material waste. To achieve near-net-shape forming while minimizing subsequent machining, this study proposes a coupled optimization strategy that considers both the elastic deformation of the die and the elastoplastic deformation of the blank. Dimensional accuracy is progressively improved through iterative compensation of the die-cavity geometry.

### 4.1. Die Compensation Procedure

**Figure 6:**
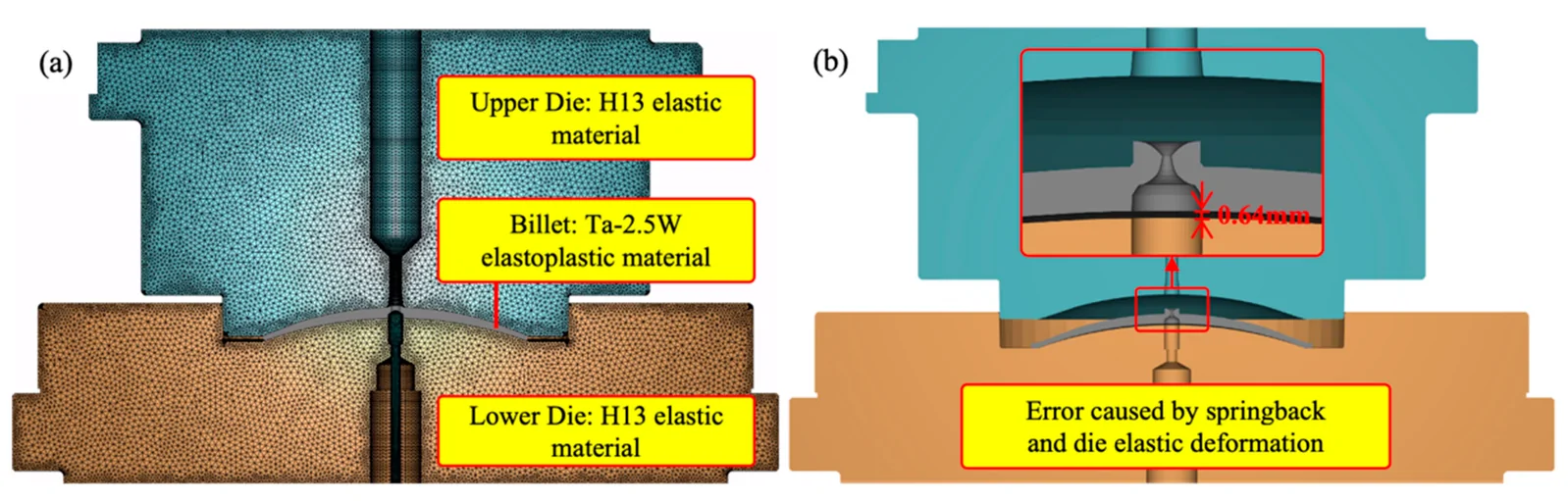
Finite element model of cold pressing for the liner: (**a**) end of loading; (**b**) end of unloading.

During cold pressing, elastic die deformation and blank springback cause a pronounced deviation of the unloaded part contour from the target contour, thereby increasing the required machining allowance, as shown in Figure 6. To address this issue, a die-correction method was developed for the precision cold forming of thin-walled liners.

The entire cold pressing simulation was performed using the commercial finite element software Forge 4.0. A 2D-dimensional elastoplastic finite element model was established for the cold forming process of the Ta-2.5W shaped charge liner. The simulation parameters has been show in Table 3. Three-node triangular elements were adopted for both the billet and dies to ensure simulation accuracy. The geometric models of the upper die, lower die, and billet were strictly constructed according to the actual structural dimensions of the experimental equipment and workpiece. Contact pairs were defined between the billet-upper die and billet-lower die interfaces. Combined shear and Coulomb friction models were used to describe interfacial frictional behavior. Automatic adaptive remeshing was activated to resolve element distortion under large plastic deformation. A mesh convergence study was carried out on three mesh densities, and the final mesh configuration was selected to balance calculation accuracy and computational efficiency.

By establishing a coupled elastoplastic finite element model of the die and blank, the proposed method predicts the unloaded part contour and the associated springback error and then iteratively adjusts the die-cavity geometry according to the spatial distribution of the contour error. This strategy reduces the number of die tryout iterations, enhances dimensional accuracy and production efficiency, and provides a technical basis for the precision forming of high-value, difficult-to-machine Ta-W alloys.

**Table 3:** Finite element simulation parameters used in the cold pressing model.

Object	Temperature (°C)	Materials	Number of Elements	Friction Model	Heat Transfer Coefficient (W·m^−1^·K^−1^)	Air Heat Transfer (W·m^−1^·K^−1^)
Billet	25	Ta-2.5W	100,000	Shear 0.30 Coulomb 0.15	11,000	20
Upper Die	25	H13	100,000			
Lower Die	25	H13	100,000			

**Figure 7:**
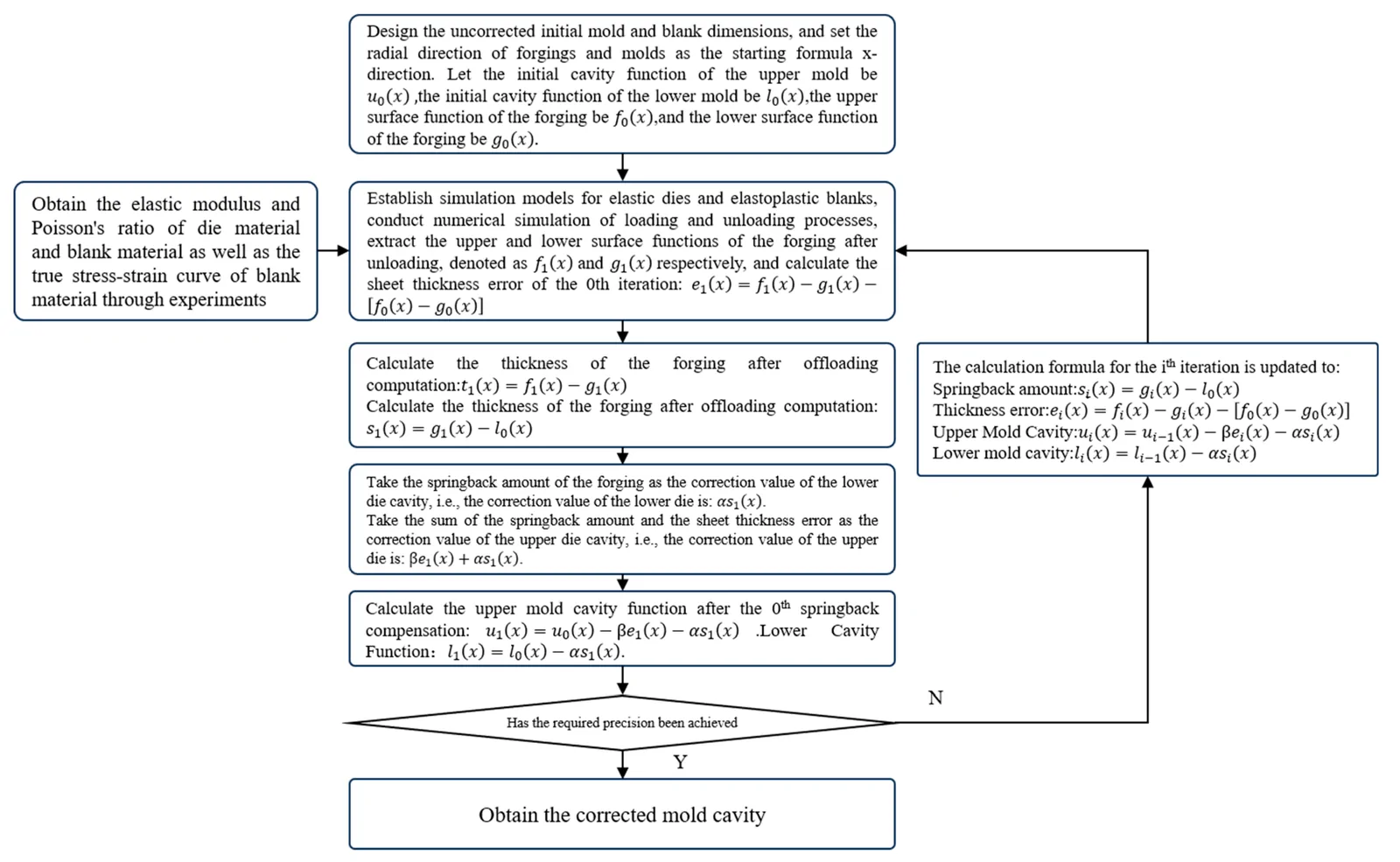
Forging error correction strategy.

To eliminate dimensional deviations arising from elastic die deformation and blank springback, a coordinated strategy for compensating the upper- and lower-die cavities was established. The iterative procedure is illustrated in Figure 7 and described below.

Step 1. Establish the initial die model: Design the theoretical die without springback correction according to the forging drawing. Discretize the forging and the die into 50 equally spaced nodes in the radial direction (defined as the initial formula x-direction), numbered j=1,2,…,50. The corresponding functions are defined respectively as follows. Initial cavity function of the upper die, $u_{0}\left(x_{j}\right)$; initial cavity function of the lower die, $l_{0}\left(x_{j}\right)$; upper surface function of the forging, $f_{0}\left(x_{j}\right)$; lower surface function of the forging, $g_{0}\left(x_{j}\right)$.

Step 2. Acquisition of material performance parameters: The mechanical properties of materials are determined through experiments, specifically including obtaining the true stress–strain curve of the blank via compression tests and acquiring that the elastic modulus of the blank is 175 GPa and Poisson’s ratio is 0.28 through tensile tests, while the elastic modulus of the die material is 210 GPa and Poisson’s ratio is 0.3.

Step 3. Establish the finite element simulation model and calculate the initial springback error: Based on the material data obtained from experiments, a finite element model for forging forming, including elastic dies and elastic-plastic blanks, is established. Numerical simulation is carried out for the loading and unloading process of the die, as shown in Figure 5. After unloading, extract the upper surface function of the forging, $f_{1}\left(x_{j}\right)$ and the lower surface function, $g_{1}\left(x_{j}\right)$. Calculate the sheet thickness error of the 0th iteration: ${e_{1}\left(x_{j}\right)=f}_{1}\left(x_{j}\right)-g_{1}\left(x_{j}\right)-\left[f_{0}\left(x_{j}\right)-g_{0}\left(x_{j}\right)\right]$.

Step 4. Calculate the springback: Calculate the springback of the forging after unloading: $s_{1}\left(x_{j}\right)=g_{1}\left(x_{j}\right)-l_{0}\left(x_{j}\right)$.

Step 5. Correct the mold cavity: Take the springback amount of the forging as the correction value of the lower mold cavity; that is, the correction value of the lower mold is $s_{1}\left(x_{j}\right)$; take the sum of the springback amount and the sheet thickness error as the correction value of the upper mold cavity, that is, the correction value of the upper mold is $e_{1}\left(x_{j}\right)+s_{1}\left(x_{j}\right)$.

Step 6. Obtain the mold cavity function after the 0th correction: Calculate the upper mold cavity function after the 0th springback compensation: $u_{1}\left(x_{j}\right)=u_{0}\left(x_{j}\right)-e_{1}\left(x_{j}\right)-s_{1}\left(x_{j}\right)$; the lower mold cavity function: $l_{1}\left(x_{j}\right)=l_{0}\left(x_{j}\right)-s_{1}\left(x_{j}\right)$.

Step 7. Iterative correction for the i-th time: Perform the i-th mold iterative correction following Steps 3 to 6. The springback calculation formula for the i-th iteration is $s_{i}\left(x_{j}\right)=g_{i}\left(x_{j}\right)-l_{0}\left(x_{j}\right)$; the sheet thickness error calculation formula is ${e_{i}\left(x_{j}\right)=f}_{i}\left(x_{j}\right)-g_{i}\left(x_{j}\right)-\left(f_{0}\left(x_{j}\right)-g_{0}\left(x_{j}\right)\right)$; the upper mold cavity calculation formula is $u_{i}\left(x_{j}\right)=u_{i-1}\left(x_{j}\right)-e_{i}\left(x_{j}\right)-s_{i}\left(x_{j}\right)$; the lower mold cavity calculation formula is $l_{i}\left(x_{j}\right)=l_{i-1}\left(x_{j}\right)-s_{i}\left(x_{j}\right)$.

Step 8. Convergence criterion and termination: Repeat Steps 3–7 until both the sheet-thickness error and the springback error are lower than the prescribed tolerance, then terminate the iteration and output the final corrected die-cavity curves.

**Figure 8:**
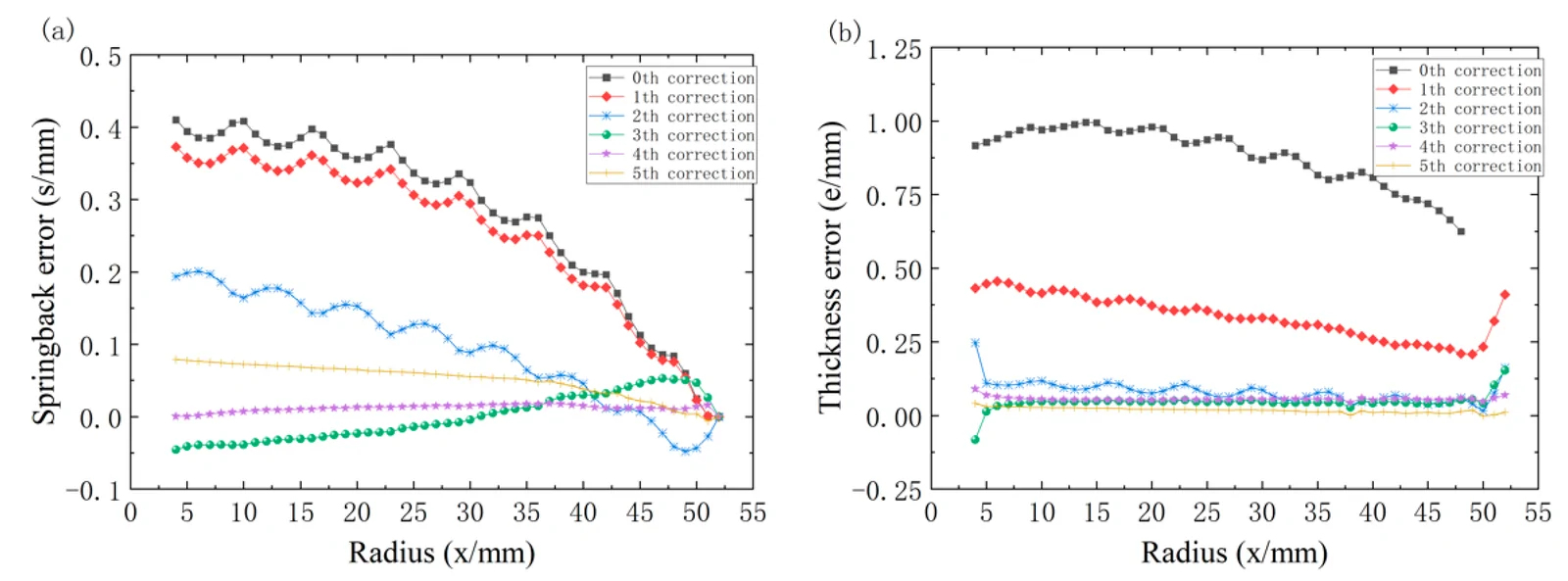
Springback error (**a**) and plate thickness error (**b**) after five corrections.

The design tolerance for both curved surfaces of the product was set to ≤0.024 mm, and the die was corrected iteratively to satisfy this requirement. Figure 8 presents the distributions of springback error and sheet-thickness error over five compensation iterations, where Figure 8a corresponds to springback error and Figure 8b to thickness error.

Both the springback error and the sheet-thickness error decrease markedly with increasing iteration number. As shown in Figure 8a, the springback error is progressively reduced to near zero after five iterations. Figure 8b shows a similar trend for the thickness error, indicating effective convergence of the compensation strategy.

**Figure 9:**
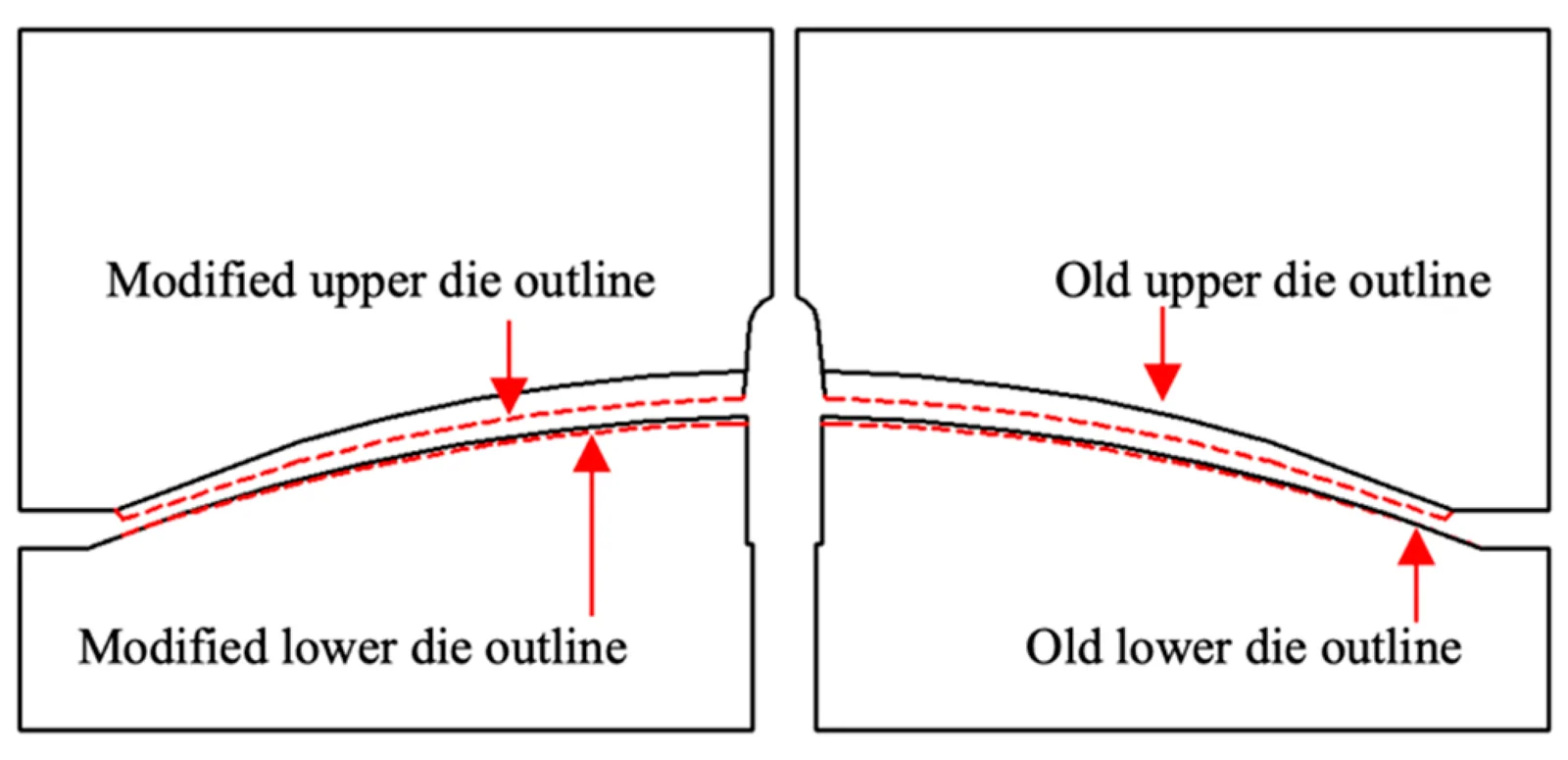
Comparison of upper and lower die contours before and after correction.

These results demonstrate that the proposed coordinated die-compensation method effectively improves the dimensional accuracy of shaped-charge liners during cold pressing. The compensated die-cavity profiles are shown in Figure 9, where the dashed curves represent the compensated profiles and the solid curves represent the original profiles. The upper-die cavity is shifted downward after compensation because the upper die undergoes upward elastic deflection during forming; the additional downward shift therefore provides the required allowance for this elastic deformation and reduces the dimensional deviation of the formed part. The compensated lower-die cavity lies below its corresponding original profile because the lower die primarily corrects springback, whereas the upper die compensates for both springback and thickness error. Accordingly, the larger compensation applied to the upper die indicates that elastic die deformation contributes substantially to the final dimensional error of the formed part.

**Figure 10:**
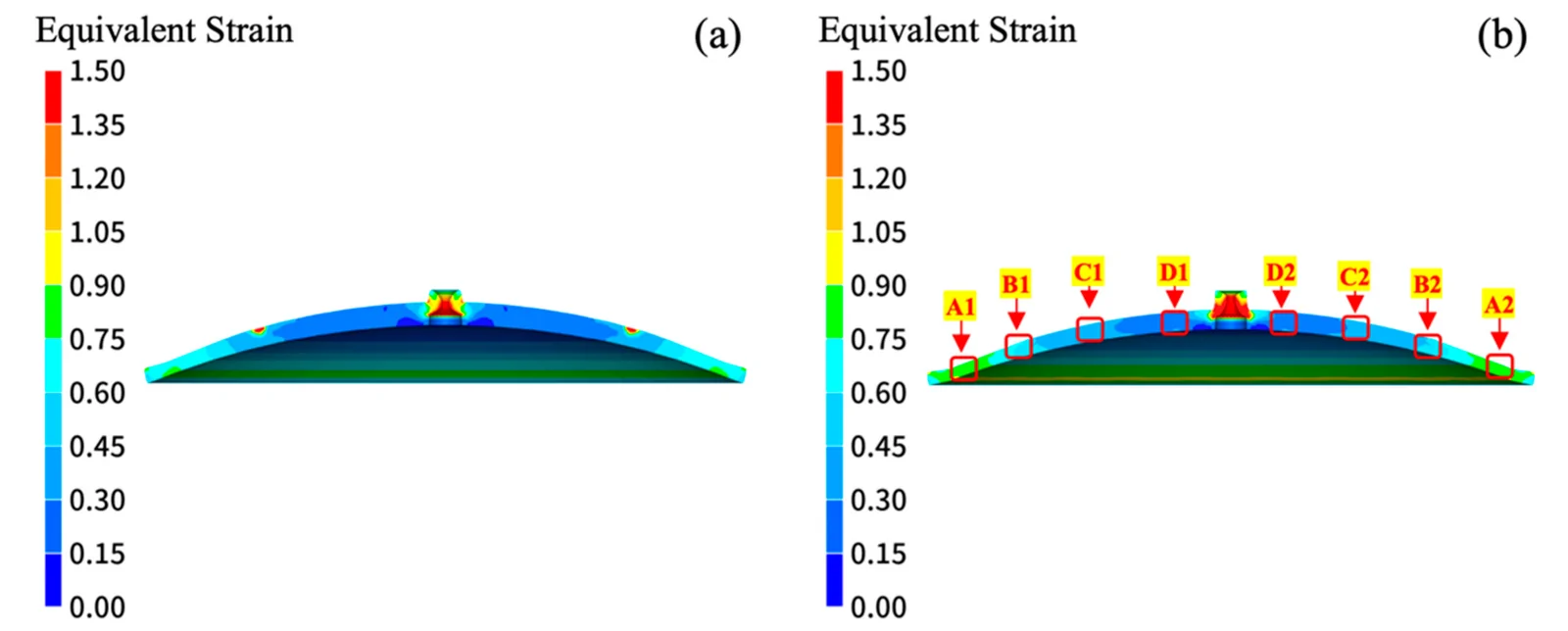
Equivalent-strain distribution before optimization (**a**) and after optimization (**b**).

Figure 10 compares the equivalent strain distributions in the part before and after die modification. After die modification, the local strain concentrations are noticeably reduced, and the equivalent strain distribution becomes more uniform. These results indicate that die modification effectively improves material flow and enhances the uniformity and compatibility of plastic deformation in the part. The equivalent strains at A1 (A2), B1 (B2), C1 (C2), and D1 (D2) are approximately 0.75, 0.55, 0.33, and 0.24, respectively. Assuming uniaxial uniform compression, these values correspond to deformation amounts of approximately 52.80%, 42.30%, 28.10%, and 21.30%, respectively. Although the degree of plastic deformation varies among the different regions, the transition in deformation between adjacent regions becomes smoother after die modification.

### 4.2. Trial Production

To further verify the engineering applicability of the coupled die-elasticity/blank-springback compensation method, cold pressing trials were performed using the corrected die profiles and the measured material parameters of the blank. Two criteria were emphasized: whether the contour error of the formed parts could be effectively suppressed and whether the microstructure after annealing was consistent with the predicted process window, thereby satisfying the quality requirements of near-net forming.

The recrystallization annealing process adopted the optimized parameters identified above, namely 1200 °C for 45 min. This condition was selected to ensure that the stored deformation energy after cold pressing was sufficient to trigger recrystallization, grain refinement, and microstructural homogenization while avoiding the crack sensitivity associated with excessive temperature or deformation.

**Figure 11:**
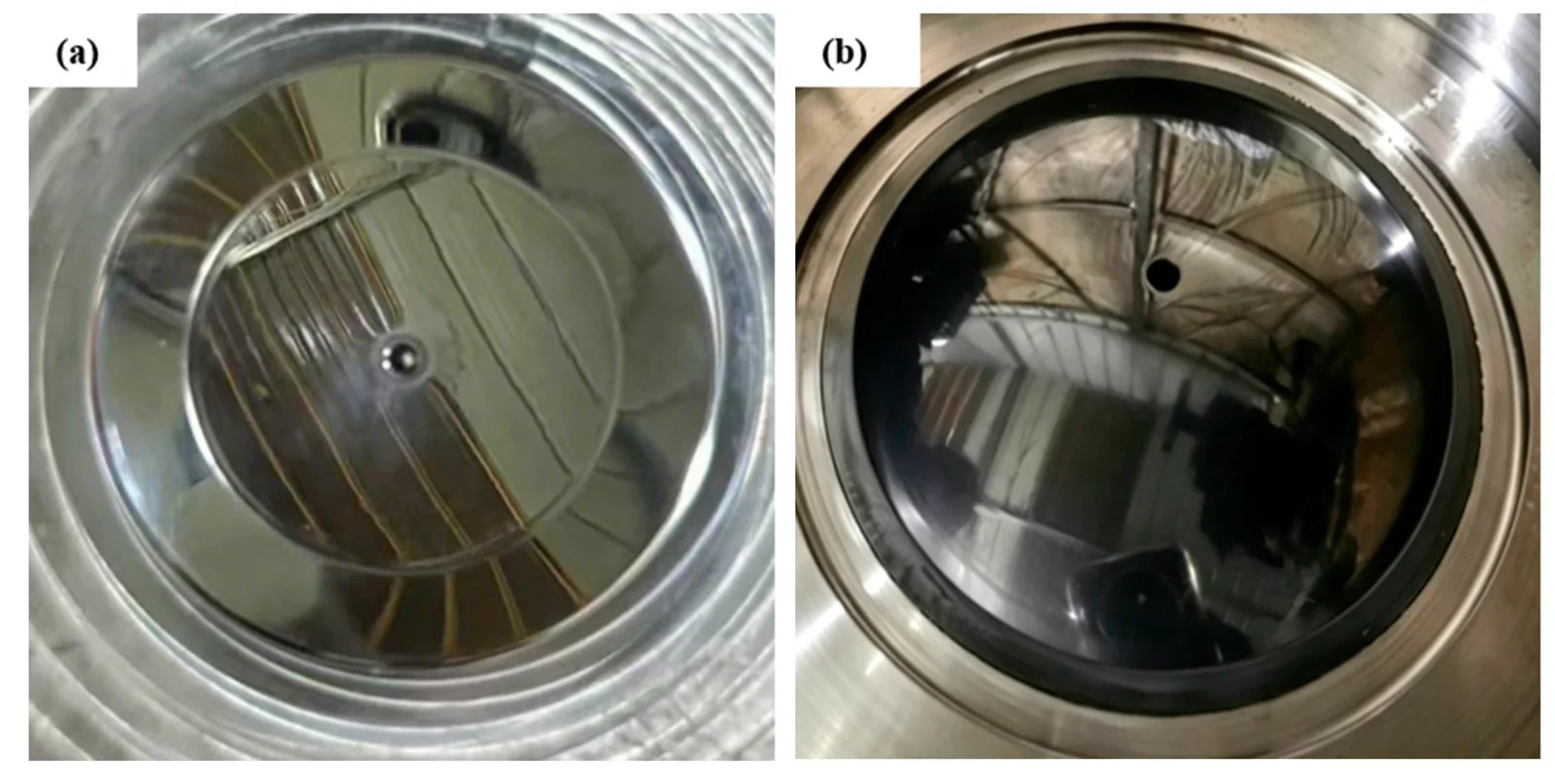

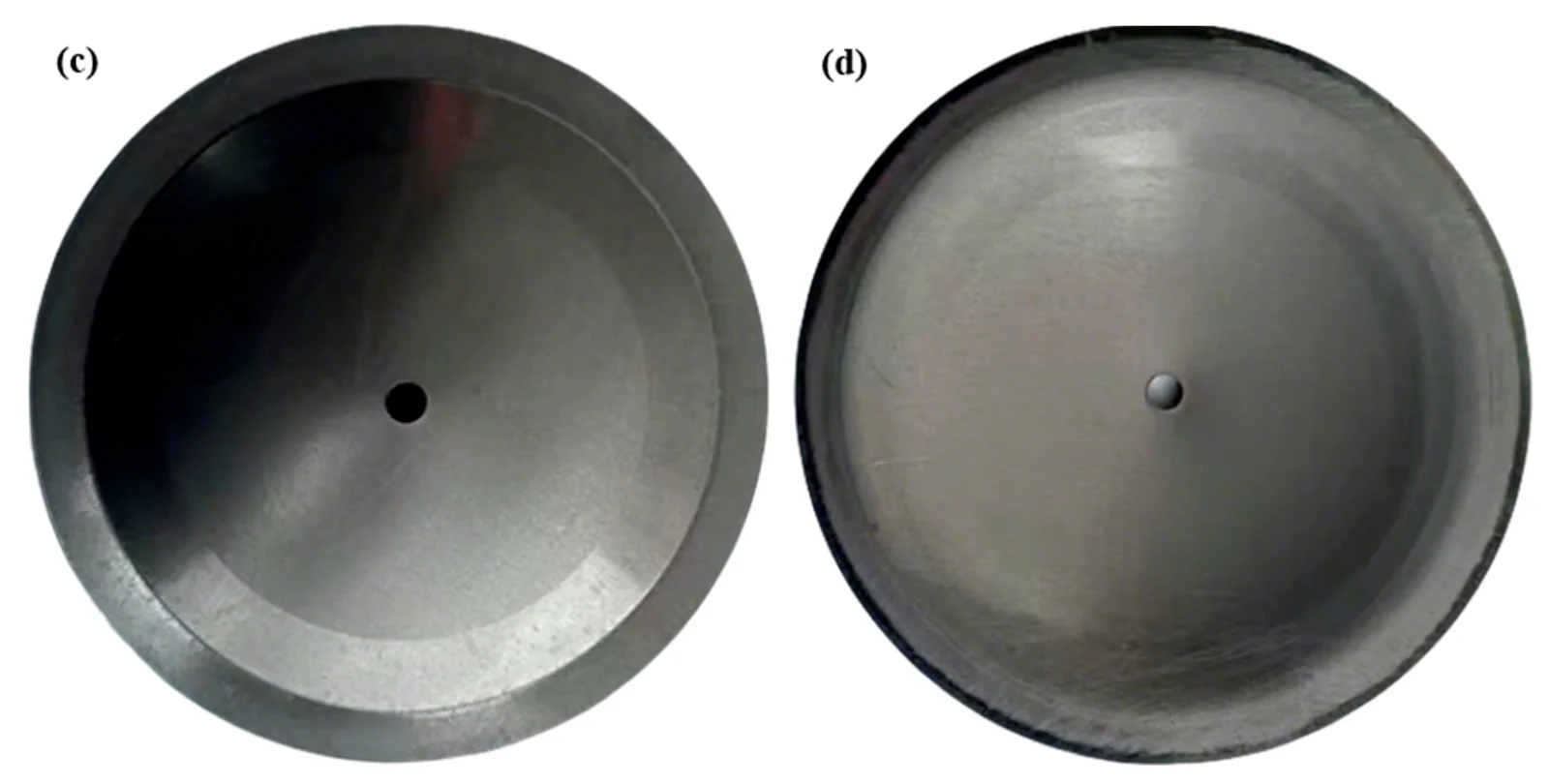
Trial-manufactured die set and formed parts for the cold pressing experiment: (**a**) upper die; (**b**) lower die; (**c**) upper surface of the part; (**d**) lower surface of the part.

Figure 11 shows the trial dies and the formed parts. The cavity surfaces of the upper and lower dies were polished and coated by PVD to obtain a mirror-like finish and to minimize the influence of die roughness on part surface quality.

**Figure 12:**
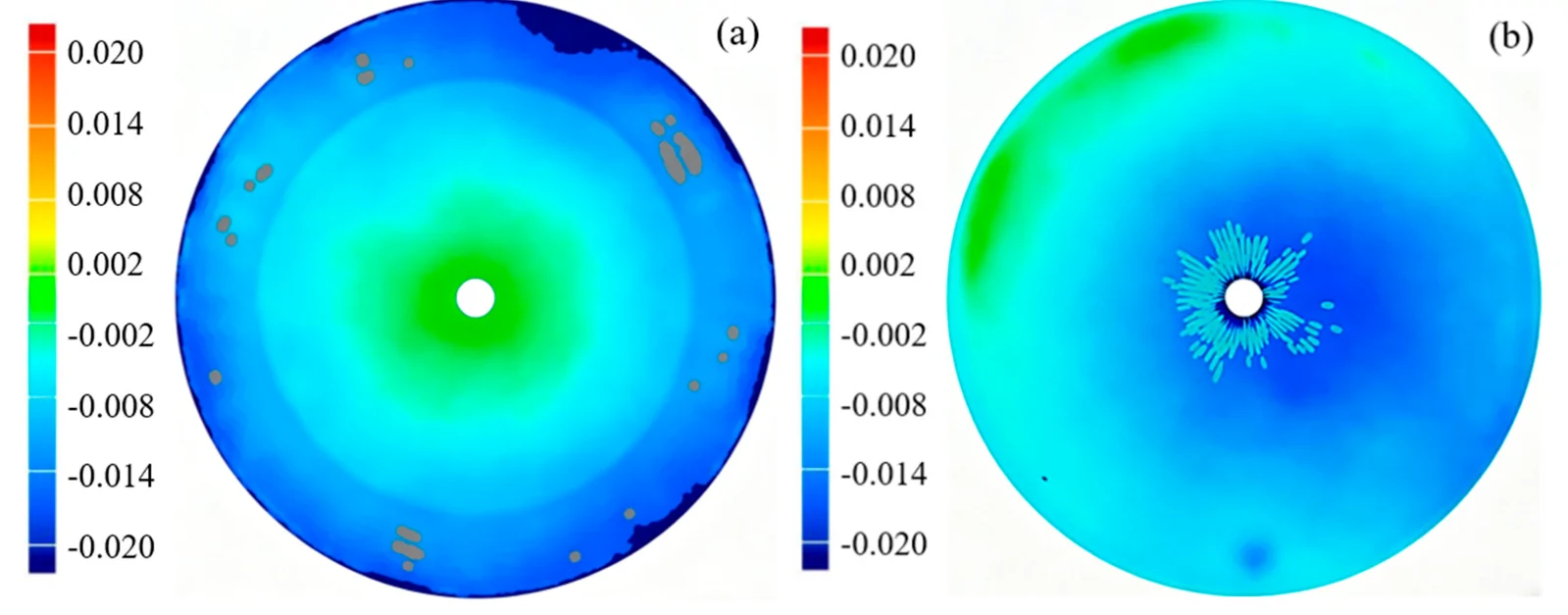
Contour comparison between cold-pressed parts and the target design: (**a**) top view; (**b**) bottom view.

Figure 12 compares the contour of the cold-pressed part with the target design contour. The modified die effectively reduces the contour deviation, and the measured contour agrees well with the design profile. In terms of geometric accuracy and machining allowance, the forming quality satisfies the basic requirements of near-net manufacturing. These results confirm that the proposed die-compensation strategy can quantitatively correct the geometric deviation caused by springback and die elastic deformation and can therefore support near-net manufacture of Ta-W shaped charge liners with minimal machining.

### 4.3. Microstructure After Cold Pressing and Recrystallization Annealing

After cold pressing, the formed specimens were subjected to recrystallization annealing to verify the consistency between the observed microstructure and the predicted process window. Based on the proposed process parameters, the parts were held at 1200 °C for 45 min and then sectioned for microstructural observation. Samples were taken from the central cross-section to reflect the spatial distribution of the microstructure within the formed part as accurately as possible.

**Figure 13:**
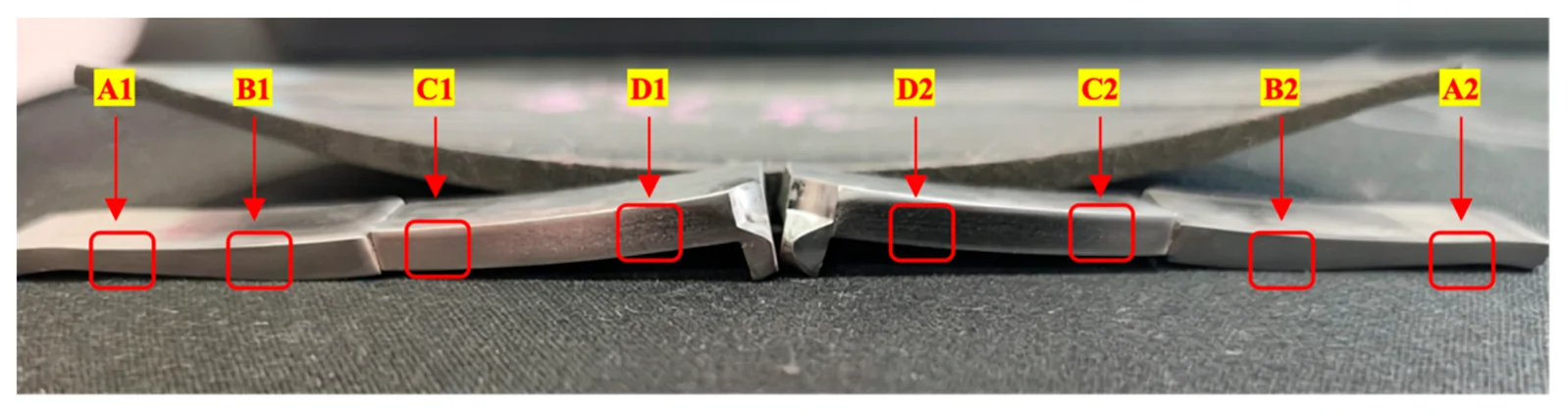
Schematic diagram of sampling locations for microstructure after recrystallization annealing.

As shown in Figure 13, several representative positions were selected on the sectioned cross-section for microstructural characterization. On the left side of Figure 13, points A1, B1, C1, and D1 extend from the outer ring toward the core, while points A2, B2, C2, and D2 are defined symmetrically on the right side. Comparing these locations makes it possible to determine whether the local deformation history during cold pressing affects grain refinement, microstructural uniformity, or the occurrence of defects such as localized coarsening and cracking after recrystallization.

**Figure 14:**
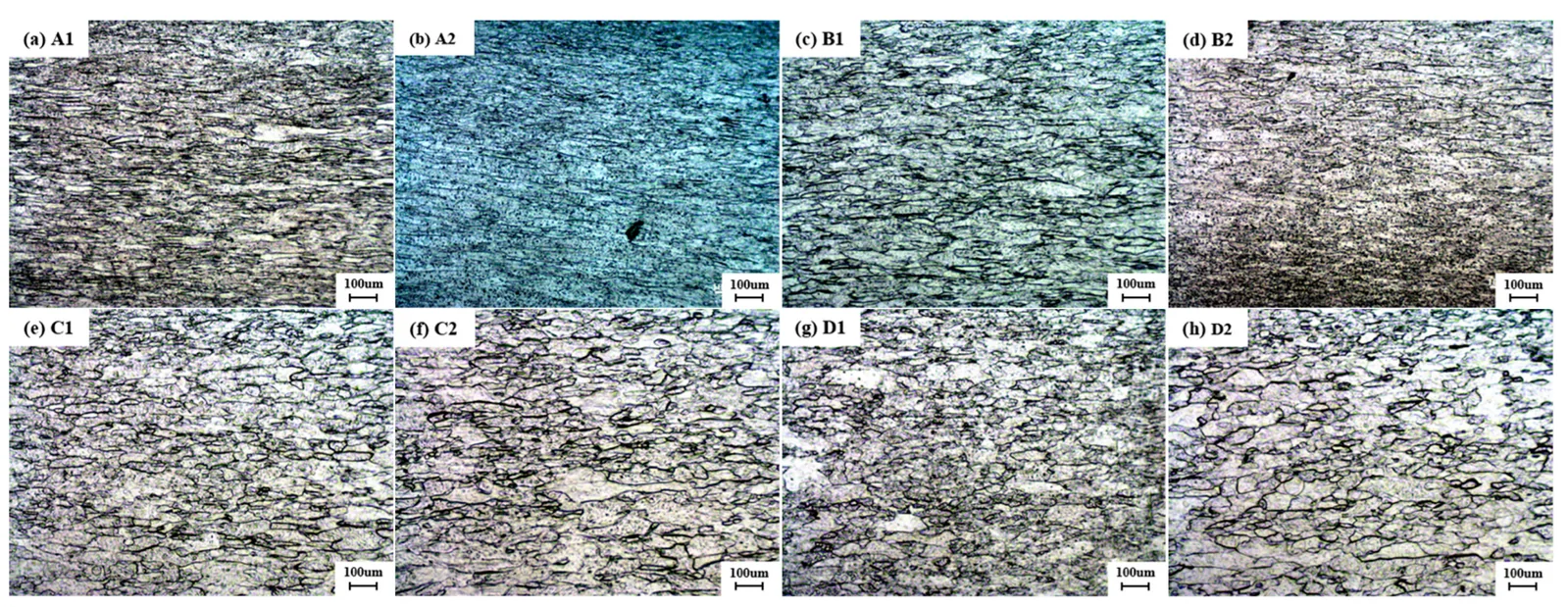
Microstructures at different sampling positions (100× magnification).

**Figure 15:**
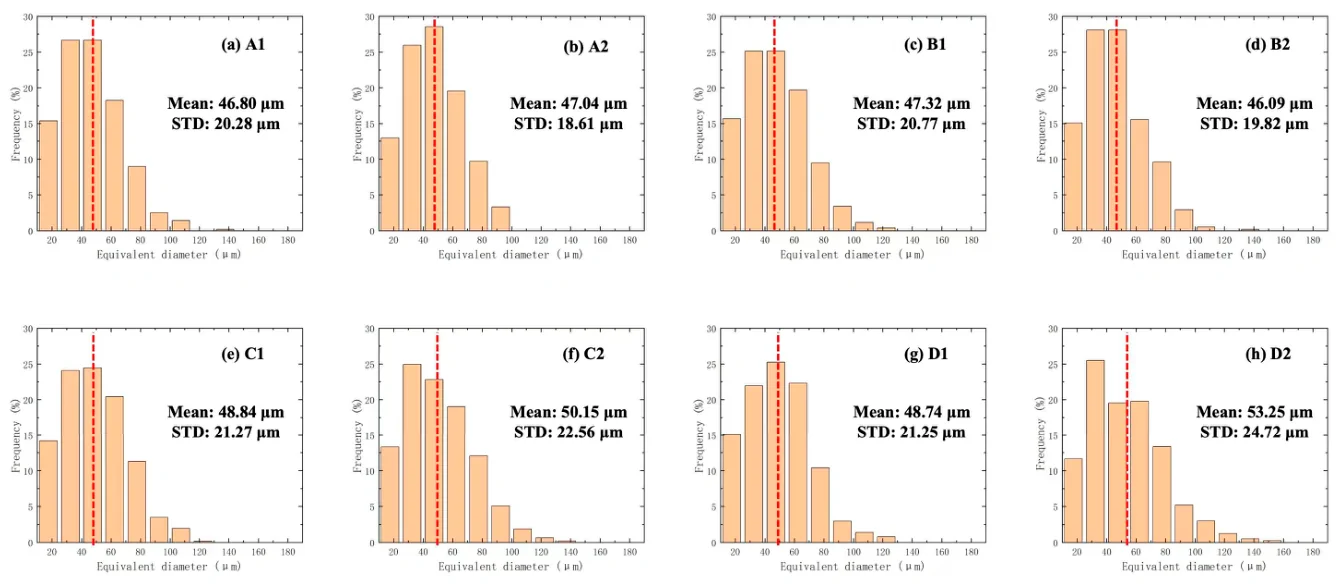
Grain-size statistical results of the microstructure at different sampling positions.

Figure 14 shows that no cracks are observed in the sampled regions, confirming good cold-forming compatibility of the part. From Figure 14a–h and Figure 15a–h, the microstructure gradually transitions from nearly equiaxed grains in the central region to a small fraction of fibrous structure toward the outer diameter, where the grains are finer. This trend is consistent with the larger local deformation near the outer diameter, which promotes more sufficient recrystallization during annealing. Although a small amount of fibrous microstructure remains near the edge, performance evaluation indicates that it still satisfies the design requirements. Moreover, locations at the same radial distance exhibit similar microstructural characteristics, indicating limited radial property variation, which is favorable for shaped charge liner applications.

## 5. Discussion

This work establishes a processing route for Ta-2.5W liners with distinctive loading paths and technical advantages compared with previously reported Ta-W fabrication strategies. Wang et al. [12] improved microstructural characteristics via cold rolling, while the current route adopts local compressive strain to promote post-forming recrystallization, realizing microstructure optimization through a different forming mechanism. Compared with the multi-pass extrusion-forging process with repeated intermediate anneals proposed by Zhan et al. [15], the present single near-net pressing combined with terminal annealing greatly simplifies the process flow and improves fabrication efficiency. Differing from the high-temperature multi-pass rolling optimization method for sheet performance proposed by Yang et al. [16], this study adopts a tailored high-temperature annealing system to achieve a uniform microstructure of formed liners. Although Kedharnath et al. [17] obtained fine-grained and high-strength Ta-W alloys through multi-axial forging, the proposed route is more targeted and applicable for low-W-content Ta-2.5W liners, forming a unique and efficient processing system for this material.

The proposed manufacturing strategy innovatively integrates near-net cold pressing, simulation-based iterative die compensation, and annealing treatments. This process framework shows promising potential within the present experimental scope, rather than excellent universality, and may be extendable to several typical liner geometries, including conical, hyperbolic, hemispherical, and variable-wall-thickness liners. Finite-element-simulation-driven die contour correction enables high-precision dimensional control and stable forming quality for the specific geometry investigated in this work.

This route demonstrates promising application prospects under laboratory conditions. It can effectively reduce the machining allowance and material loss of high-cost Ta-W raw materials and avoid excessive die-trial iterations to shorten the process-development cycle. The established simulation–measurement–compensation closed-loop manufacturing system delivers high forming accuracy and favorable process controllability for the tested Ta-2.5W liner case in this study, and its feasibility for pilot-scale or mass-production scenarios remains to be further verified. Under the current experimental conditions, appropriate tuning of blank parameters, lubrication conditions, mesh resolution, die-compensation magnitude, and annealing regimes according to sheet thickness and component size may help obtain high-quality Ta-2.5W liners for similar geometries. This work provides a feasible technical reference for developing manufacturing routes of high-performance thin-walled Ta-W components.

It should be noted that only one specimen was formally characterized for each deformation-annealing condition in this work. Multiple metallographic fields at different positions of each sample were analyzed to mitigate the influence of local heterogeneity, whereas the statistical probability of crack occurrence cannot be derived from the limited sample quantity. This study mainly focuses on revealing microstructural evolution and crack-generation mechanisms under near-net cold pressing. Further experiments with more parallel specimens will be required to obtain statistically reliable process windows in future investigations.

## 6. Conclusions

This study systematically investigated the initial material properties, cold compression behavior, annealing microstructure evolution, and die-compensation strategy for the near-net-shape forming of Ta-2.5W alloy liners. Based on microstructural characterization, mechanical testing, numerical simulations, and trial production, the following conclusions can be drawn:

(1) Ta-2.5W alloy exhibits an ultimate tensile strength of approximately 350 MPa, an elongation at fracture of 35.5%, an elastic modulus of 176 GPa, and a Poisson’s ratio of 0.28. The grain size varies only slightly among the observation directions, indicating a relatively uniform microstructure and weak anisotropy, which are favorable for cold forming. The room-temperature plastic-flow constitutive model calibrated using the compressive stress–strain curves can be combined with the measured elastic parameters to provide reliable material input for the numerical simulation and preliminary process exploration of Ta-W alloy cold forming.
(2) A preliminary processing range suggested by the present single-specimen screening experiments was obtained for the cold-compressed Ta-2.5W alloy regarding its recrystallization-related annealing treatment. At a constant annealing temperature, increasing the deformation level promotes grain refinement and reduces the fraction of the banded microstructure; at a constant deformation level, increasing the annealing temperature initially promotes grain refinement but subsequently causes grain coarsening. At annealing temperatures of 1250 °C or higher, specimens subjected to 40% deformation become susceptible to through-thickness cracking. Considering both structural integrity and microstructural uniformity, a preliminary processing range suggested by the present single-specimen (n = 1) screening experiments covers 20–40% cold deformation followed by annealing at 1200 °C.
(3) For thin-walled shaped-charge liners, a die-compensation model accounting for elastic die deformation and blank springback was established. Iterative correction markedly reduced both springback and thickness errors and enabled trial production using the proposed process and corrected dies. The resulting parts conformed to the target contour, remained crack-free after annealing, and exhibited a generally uniform microstructure in the radial direction, with moderate grain refinement near the outer diameter. The proposed near-net-shape cold-forming route therefore provides a practical basis for the precision manufacture of Ta-W shaped-charge liners.

## Figures and Tables

**Table 1:** Chemical composition of the as-received Ta-2.5W alloy (wt.%).

W	O	N	C	H	Fe	Ni	Ta
2.47	0.042	0.021	0.018	0.001	0.023	0.008	Bal.

**Table 2:** Experimental matrix for different deformation levels and recrystallization annealing temperatures.

Sample No.	Deformation Amount	Annealing Temperature	Sample No.	Deformation Amount	Annealing Temperature
1	10%	1150 °C	9	10%	1250 °C
2	20%		10	20%	
3	30%		11	30%	
4	40%		12	40%	
5	10%	1200 °C	13	10%	1300 °C
6	20%		14	20%	
7	30%		15	30%	
8	40%		16	40%	

## Data Availability

The original contributions presented in this study are included in the article. Further inquiries can be directed to the corresponding authors.

## DURC Statement

The present research focuses exclusively on the near-net cold forming, annealing treatment, and numerical simulation of Ta-2.5W alloy shaped charge liners. All experiments involve only conventional metallic materials and standard mechanical testing (tensile, compression, and metallographic examination) under benign laboratory conditions. No explosive, energetic, or hazardous substances are synthesized, handled, or used in this work. The computational aspects are limited to finite-element modeling of elastoplastic deformation, springback, and die compensation, which serve solely to improve dimensional accuracy and process efficiency for thin-walled components. Therefore, this study poses no threat to public health, biosecurity, or national security. The authors acknowledge that the computational methods and forming techniques described herein could, in principle, be adapted to other engineering alloys, but all procedures are conducted within the framework of responsible material processing. We confirm that all necessary precautions have been taken to prevent potential misuse, and we strictly adhere to national and international DURC regulations. We advocate for ethical conduct, regulatory compliance, transparent reporting, and the beneficial application of our findings.
